# In Vitro Anti-Proliferative and Cisplatin-Modulating Effects of *Cedrus libani* A. Rich Leaf Essential Oil on H460 and HT-29 Human Cancer Cell Lines

**DOI:** 10.3390/ijms27209044

**Published:** 2026-10-12

**Authors:** Hicham Younes, Bilal Azakir, Rania Zaarour, Nathalie Hayeck, Jamilah Borjac

**Affiliations:** 1 Faculty of Science, Beirut Arab University, Debbieh, Beirut 11072809, Lebanon; hicham-younes@hotmail.com; 2 Molecular and Translational Medicine Laboratory, Faculty of Medicine, Beirut Arab University, Beirut 11072809, Lebanon; b.azakir@bau.edu.lb; 3 Thumbay Research Institute for Precision Medicine, Gulf Medical University, Ajman 4184, United Arab Emirates; dr.rania@gmu.ac.ae; 4 Department of Physical Sciences, Lebanese American University, Beirut 11022801, Lebanon; nathalie.hayeck@lau.edu.lb

**Keywords:** anticancer, *C. libani*, cisplatin, reactive oxygen species, nuclear morphology

## Abstract

*Cedrus libani* A. Rich is a coniferous tree native to the eastern Mediterranean region that is known for exhibiting pharmacological properties. In the present study, we investigated the anti-proliferative effect of *C. libani* leaf essential oil (EO) against H460 and HT-29, as well as evaluated its chemo-modulatory effect with cisplatin (CIS). The EO chemical profile was analyzed via GC-MS. Cell viability was assessed using MTT, while trypan blue exclusion and Annexin V/Propidium Iodide staining were employed to detect cell death. Colony biomass and wound closure were evaluated using crystal violet staining and a scratch assay, respectively. Nuclear morphology and reactive oxygen species (ROS) were assessed using DAPI staining and an NBT assay, respectively. EO and CIS individually reduced cell viability in a time- and concentration-dependent manner with combination treatments demonstrating synergistic/additive/antagonistic interactions depending on the combination ratio, cell line and time point. EO alone does not exhibit significant cell death, whereas CIS demonstrated significant apoptosis and necrosis. The combination (IC_50_:IC_50_) enhanced cell death, particularly in HT-29. Both EO and CIS reduced colony biomass and wound closure, with the combination showing higher inhibitory effect on colony biomass in both cell lines and enhanced wound closure inhibitory effect in HT-29 but reduced effect in H460. CIS and combination caused more notable nuclear alterations compared to EO alone, whereas an early increase in NBT reduction followed by a subsequent decrease was observed for individual treatments as well as the combination. This study provides insights into the antiproliferative and chemo-modulatory potential of *C. libani* leaf essential oil, although further studies are needed to elucidate the underlying molecular mechanisms.

## 1. Introduction

According to Global Cancer Statistics 2022, there was an estimate of 20 million new cancer cases and 9.7 million cancer deaths, with lung and colorectal cancers being among the most frequently diagnosed and leading causes of cancer-related deaths [1].

Chemotherapy is an effective widespread approach of treating malignancies [2]. Cisplatin (cis-diammine-dichloro-platinum II) is one of the most efficient and the first metal-based chemotherapeutic agents used in the treatment of a wide range of solid tumors [3]. The most widely recognized modes of action of cisplatin are the formation of DNA-platinum adducts and the generation of ROS, thus inducing cellular and nuclear damage [4]. In many cases, combination therapies incorporating natural products with cisplatin has emerged as a promising strategy for enhancing antitumor efficacy [5].

Lebanese Cedar (*Cedrus libani* A. Rich.), belonging to the Pinaceae Family, is a coniferous tree native to the mountains of the Mediterranean region particularly in Lebanon, Syria and Turkey [6]. Diverse preparations from *C. libani* have been traditionally used for the treatment of ailments including inflammatory, microbial, and respiratory conditions [6,7,8]. Despite its interesting folk applications, *C. libani* remains a relatively understudied tree species, particularly with respect to its biomedical potential [9]. Nevertheless, several ethnomedical, phytochemical and bioactivity studies have investigated various parts of the tree, reporting anti-infectious, anti-inflammatory, antioxidant, and preliminary anticancer activities, highlighting its promise as a source of bioactive compounds [10,11].

Recently, attention has focused on the essential oil of *C. libani*, which is known for exhibiting promising biological activities that are largely attributed to its diverse composition of terpenoid-based constituents [6]. More particularly, the *C. libani* wood oil, due to its major constituents himachalol and himachalene, have shown potent anti-proliferative activity against several cancer cell lines including drug-resistant ones, as well as demonstrating a more pronounced effect when combined with standard drugs [10,12,13,14]. On the other hand, the oil derived from the leaves of the tree is less well documented, particularly in terms of its anti-proliferative and combinatorial effects. Nevertheless, the leaves may bear potential due to their enrichment in germacrene D and ß-Caryophyllene, as previously reported [6,15]. The present study investigates the anti-proliferative and cytotoxic effects of the essential oil derived from the leaves of *C. libani* against non-small cell lung cancer (NSCLC) cell line H460 and colorectal adenocarcinoma HT-29, with a particular emphasis on its combined effect with cisplatin.

## 2. Results

### 2.1. Chemical Profiling of EO

Based on NIST14 mass spectral library matching, GC-MS analysis putatively identified a total of 41 compounds, of which the monoterpene hydrocarbons “MH” represented the largest class, accounting for 29.16%. However, the most abundant individual compounds identified within the EO was caryophyllene oxide, belonging to class of oxygenated sesquiterpenes (OS), at 21.16% (Table 1).

### 2.2. EO Decreases Viability in H460 and HT-29 Cells

**Figure 1:**
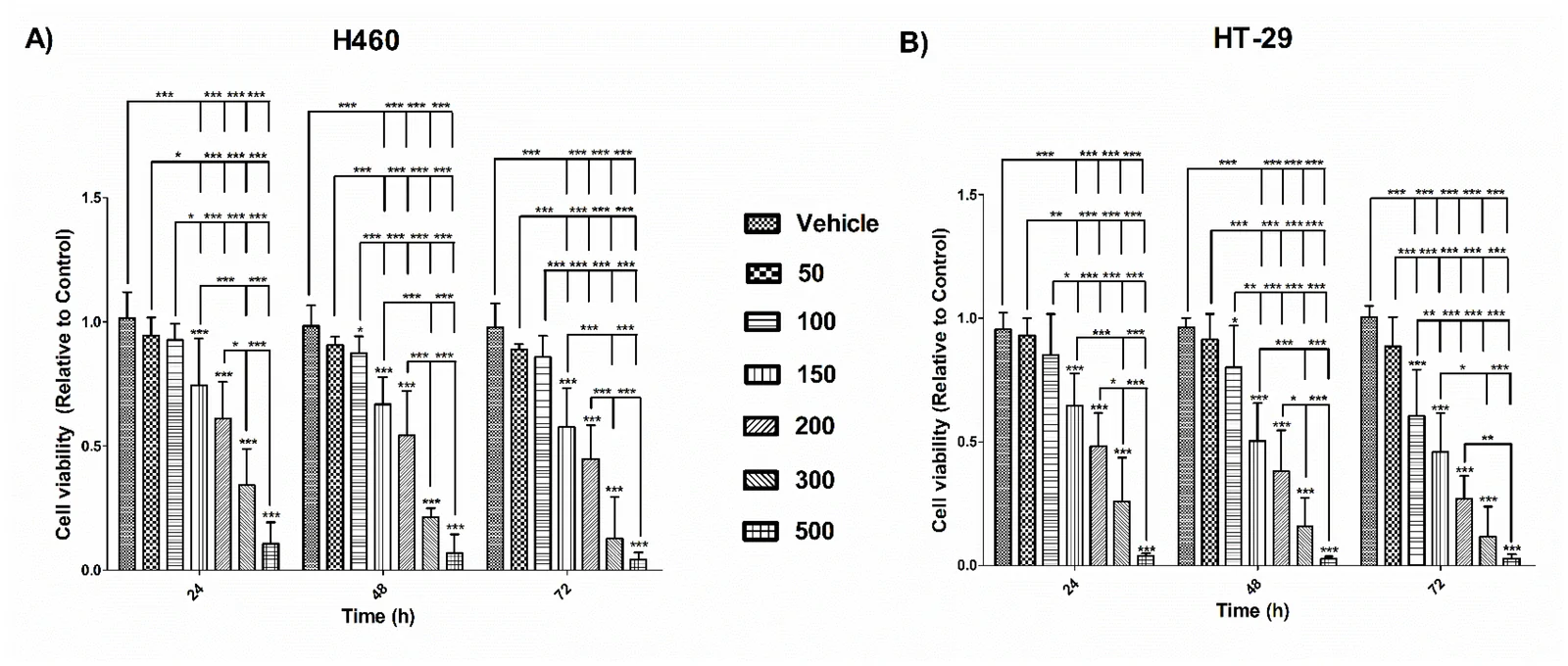
Effect of various concentrations (in μg/mL) of *C. libani* leaf-derived EO on cell viability of H460 (**A**) and HT-29 (**B**) at 24, 48 and 72 h as determined via an MTT assay. Data presented is the mean ± SD from three different experiments. Statistical evaluation was conducted using a hierarchical approach: a three-way ANOVA (cell × time × conc.) and two-way ANOVAs (time × conc. within each cell line) evaluated global main effects and interactions, while one-way ANOVAs served as post-hoc slice tests for multi-dose comparisons within individual time points. Group means were compared using Tukey’s multiple comparisons test. Asterisks on bars denote significance relative to control. Asterisks on connecting lines represent significant differences across specific treatment groups (including the DMSO vehicle group). No significant differences were observed between the untreated and vehicle controls. (*), (**), and (***) correspond to *p* < 0.05, 0.01, and 0.001, respectively.

A time- and concentration-dependent decrease in the viability of H460 and HT-29 cell lines was observed in response to EO (Figure 1), revealing a main effect of concentration (*p*-value < 0.001, F (12, 387) = 3.797, ηp^2^ = 0.891), time (*p*-value < 0.001, F (2, 387) = 10.086, ηp^2^ = 0.05) and cell (*p*-value < 0.001, F (1, 387) = 12.754, ηp^2^ = 0.032). Across all time-points, HT-29 exhibited slightly more responsiveness to EO than H460, as reflected by the IC_50_ values at 72 h of ~132 µg/mL and 165 µg/mL for HT-29 and H460, respectively, although the response kinetics between both cell lines remains parallel and consistent across time points and concentrations (*p*-value > 0.05 for all interaction terms) (Figure 1). Similar to EO, CIS treatment (Figure S1) revealed a main effect of concentration (*p*-value < 0.001, F (9, 318) = 243.842, ηp^2^ = 0.873), time (*p*-value < 0.001, F (2, 318) = 207.442, ηp^2^ = 0.566) and cell (*p*-value < 0.001, F (1, 318) = 205.124, ηp^2^ = 0.392), with HT-29 being several times more resistant than H460, yielding an IC_50_ values at 72 h of ~10.1 µg/mL and 1.87 µg/mL, respectively. However, CIS has a more complex kinetic profile characterized by significant interaction across all factors (*p*-value = 0.007, F (10, 318) = 2.489, ηp^2^ = 0.073). The IC values for EO and the corresponding non-linear regression dose-response curves, alongside their goodness of fit (R^2^), are depicted in Table 2 below and Supplementary Figure S2, respectively. Similarly, those for CIS are presented in Supplementary Table S1 and Figure S3.

### 2.3. Combinatory Effect of EO and CIS on H460 and HT-29 Viability

**Figure 2:**
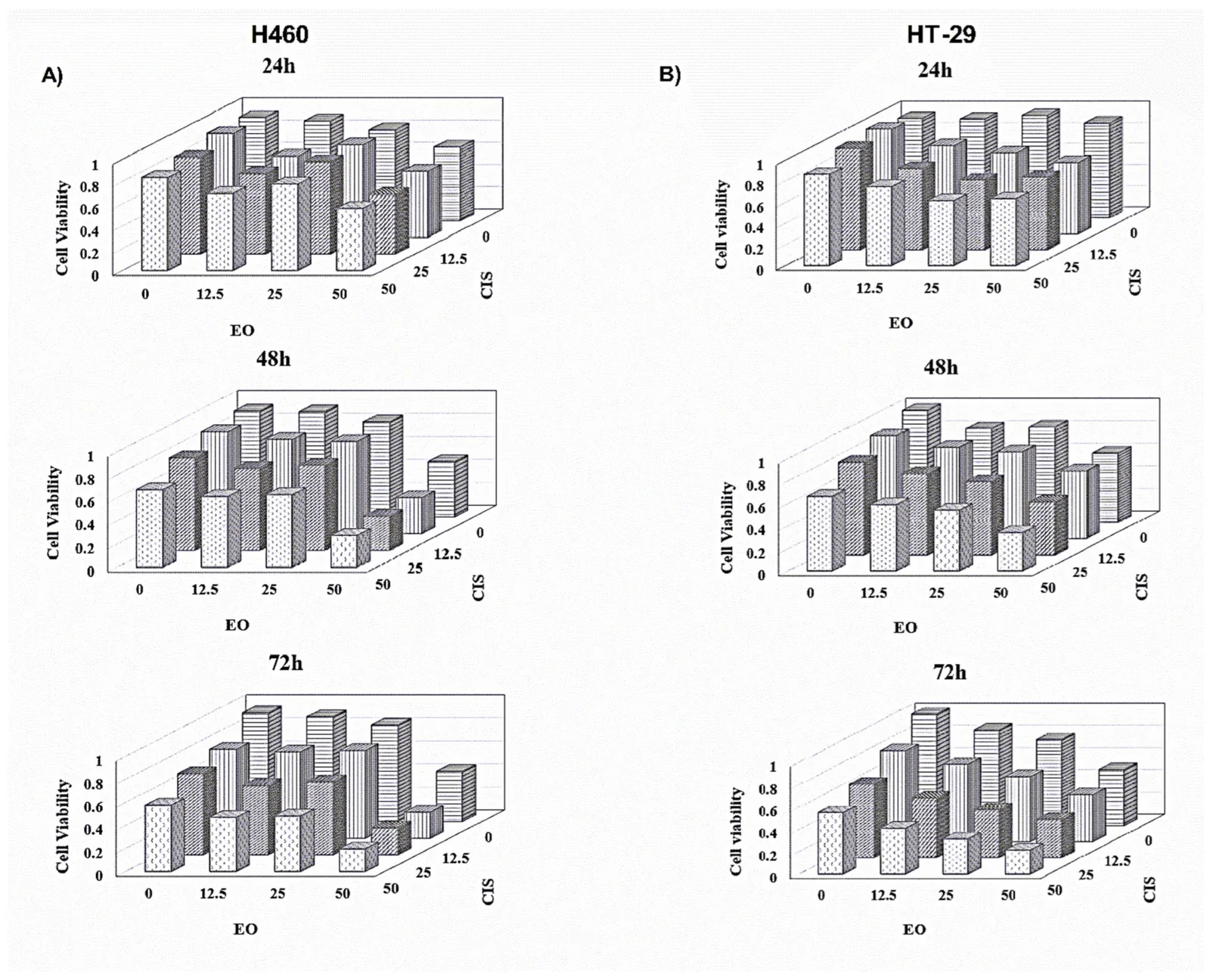
Effect of EO:CIS combination treatment on H460 (**A**) and HT-29 (**B**) evaluated using 3 × 3 factorial matrix. Cell viability was determined at 24, 48 and 72 h using an MTT assay. Data points represent mean values from independent experiments (n = 3). Statistical evaluation was conducted using a two-way ANOVA (EO × CIS) and three-way ANOVA (time × EO × CIS) to evaluate the interaction dynamics across time points.

In the H460 cell line, three-way ANOVA demonstrated significant main effects for each of EO and CIS and a significant interaction between both agents (EO × CIS, *p*-value = 0.004, F (9, 229) = 2.763, ηp^2^ = 0.098). However, the three-way interaction was not significant (EO × CIS × time, *p*-value > 0.05, F (18, 229) = 0.594, ηp^2^ = 0.045). In fact, while IC_12.5_ EO exhibited a transient mild synergy at 24 h with all CIS combinations (1.15 < Q < 2), which shifted towards an additive-antagonistic response at 48 and 72 h, IC_25_ and IC_50_ EO concentrations maintained a nearly consistent interaction across all time points; antagonistic for IC_25_ and additive for IC_50_. In HT-29, ANOVA did not yield a significant three-way interaction term (EO × CIS × time, *p*-value > 0.05, F (18, 542) = 1.255, ηp^2^ = 0.04). Despite that non-significant overall model, time-dependent evaluations and Q values revealed an acute synergistic phase at 24 h (EO × CIS at 24 h, *p*-value = 0.002, F (9, 156) = 3.053, ηp^2^ = 0.15) in all tested concentrations and a temporal shift where the interaction transitioned towards an additive response as the exposure duration extended (Figure 2 and Table 3).

### 2.4. Effect on the Proportion of Viable and Dead Cells of H460 and HT-29

**Figure 3:**
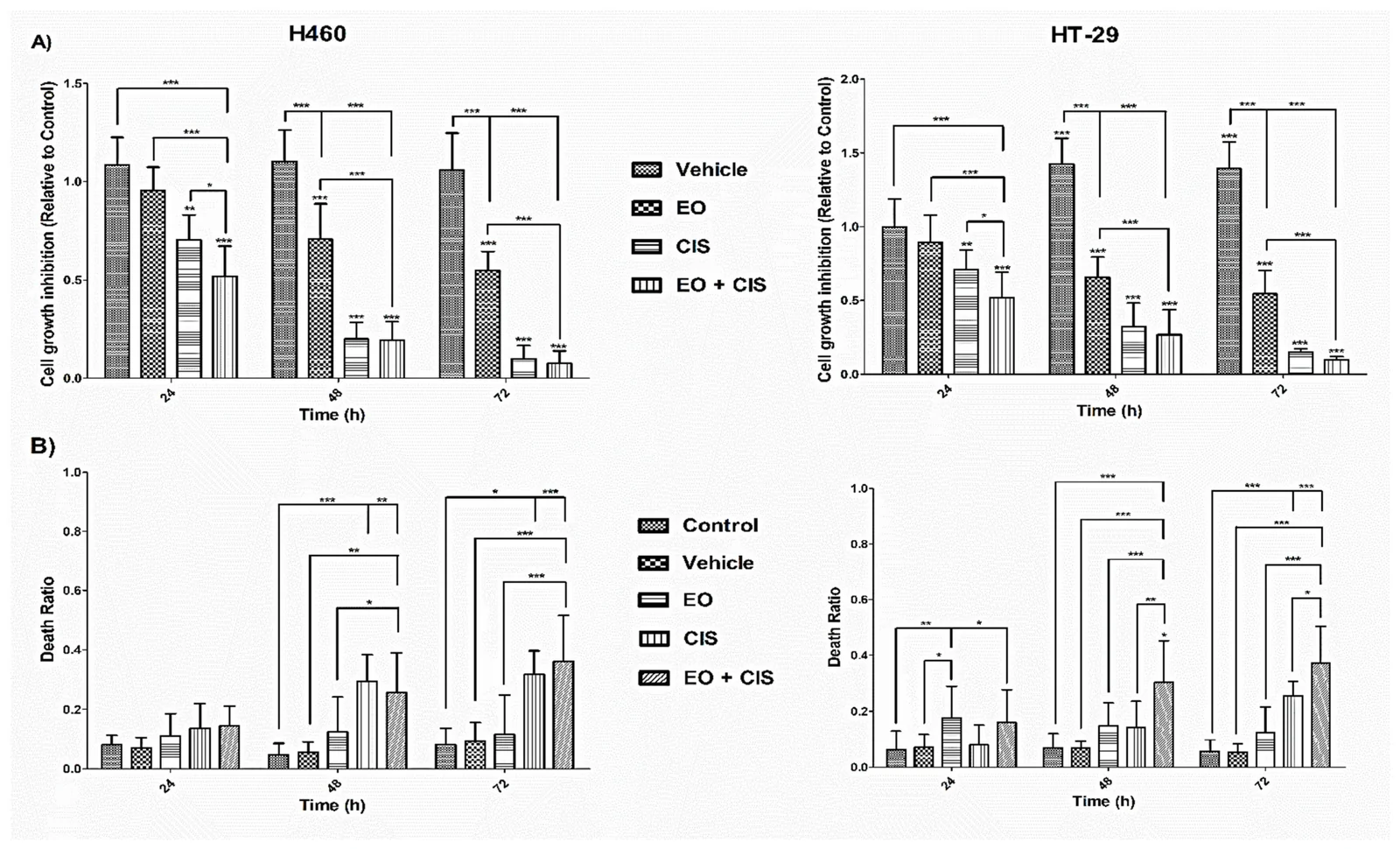
Effect of EO and CIS, individually and in combination (IC50:IC50 at 72 h), on ratio of viability (**A**) and death (**B**) of H460 and HT-29, as determined via the Trypan Blue Exclusion Assay at 24, 48 and 72 h. Data presented is the mean ± SD from three different experiments. Statistical analysis was performed using two-way ANOVA (EO × CIS) and three-way ANOVA (time × EO × CIS) for evaluating interaction dynamics across time points, while one-way ANOVAs served as post-hoc slice tests for multi-group comparisons within individual time points. Group means were compared using Tukey’s multiple comparisons test. In (**A**), the asterisks on bars denote significance relative to the untreated control. The asterisks on connecting lines represent significant differences across specific treatment groups (including the DMSO vehicle group). In (**B**), the asterisks on connecting lines represent significant differences across specific treatment groups (including the untreated and the DMSO vehicle group). (*), (**), and (***) correspond to *p* < 0.05, 0.01, and 0.001, respectively.

A significant EO × CIS interaction was observed for cell growth inhibition in both H460 (*p*-value < 0.001, F (2, 112) = 14.020, ηp^2^ = 0.2) and HT-29 (*p*-value < 0.001, F (2, 134) = 9.204, ηp^2^ = 0.121). Specifically, each individual treatment significantly reduced cell viability in both cell lines starting 24 h with a more pronounced effect at 48 and 72 h. However, cell viability was further reduced by the EO:CIS combination, as early as 24 h, compared to each individual treatment. On the other hand, the impact of cell death was particularly notable in CIS-treated cells, especially at 48 and 72 h in both cell lines, whereas the EO-treated group showed no significant cell death effect across all time intervals in H460 cells and a modest cell death response at 24 h in HT-29. The combination was shown to exhibit a higher level of cell death compared to each individual treatment, particularly in HT-29 cells (Figure 3).

### 2.5. Effect on Apoptosis/Necrosis of H460 and HT-29

EO alone did not exhibit any significant effect on either cell line. In contrast, an effect was observed with CIS and EO:CIS combination, particularly at 48 and 72 h.

In H460, necrosis was the predominant cell death mechanism in response to CIS reaching 29.55 and 30.76% by 48 and 72 h, respectively (CIS × time, *p*-value < 0.001, F (2, 30) = 17.737, ηp^2^ = 0.542). While the combination showed a significantly antagonizing effect at 48 h (EO × CIS, *p*-value = 0.012, F (1, 10) = 9.366, ηp^2^ = 0.484), it reached comparable or slightly higher level to CIS by 72 h, albeit not significantly (EO × CIS, *p*-value > 0.05).

**Figure 4:**
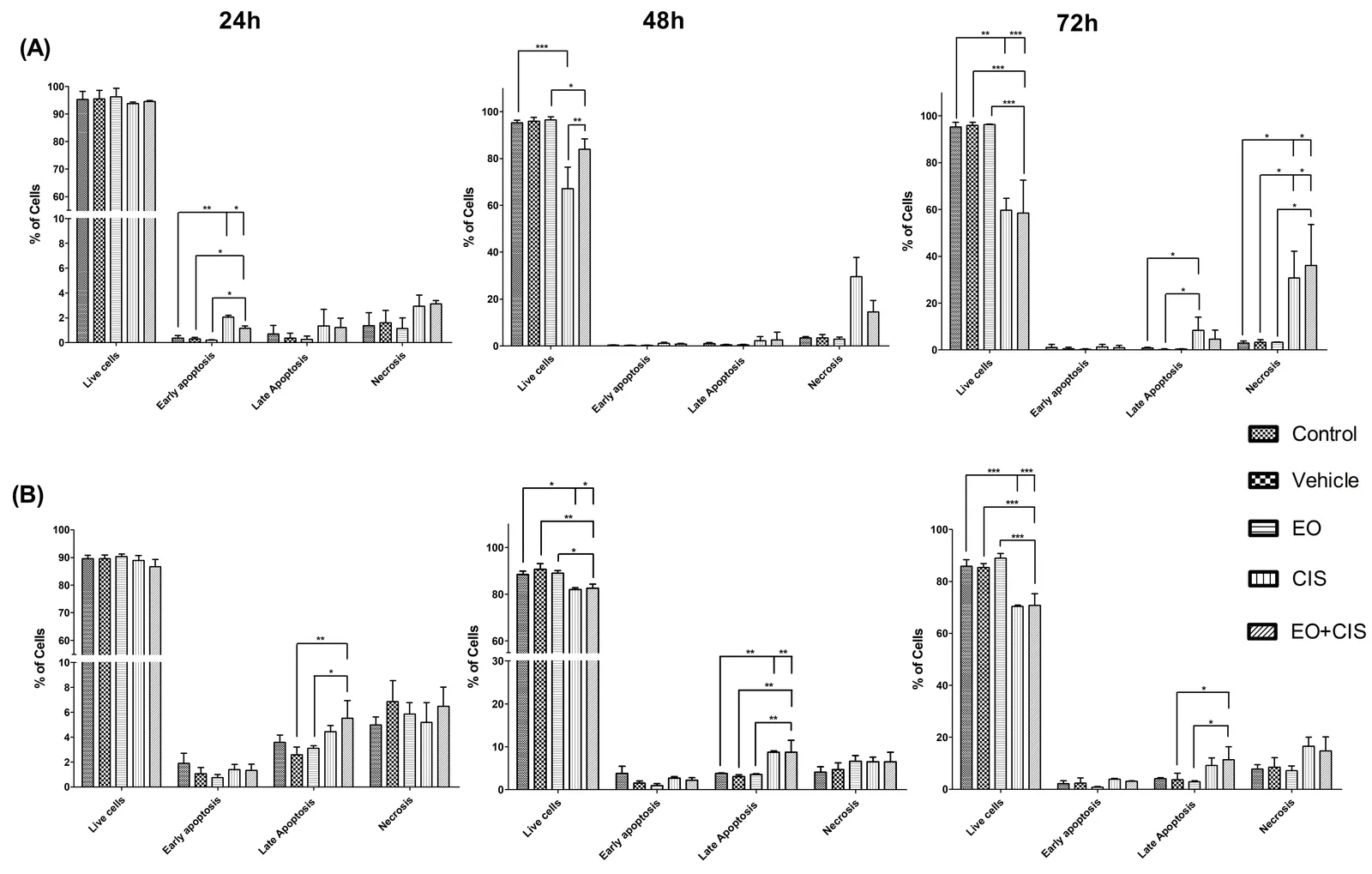
Annexin V-APC/PI flow cytometry analysis of H460 (**A**) and HT-29 (**B**) obtained at 24, 48 and 72 h in response to EO, CIS and their combination (IC50:IC5072h). The proportion of necrotic (Annexin V−/PI+; upper left), live cells (Annexin V−/PI−; lower left), early apoptotic cells (Annexin V+/PI−; lower right) and late apoptotic cells (Annexin V+/PI+; upper right) were determined and quantified. Data presented is the mean ± SD from three independent experiments (n = 3). Statistical significance was conducted using three way ANOVA (EO × CIS × time) for each quadrant and two-way ANOVA (EO × CIS) for each time point within each quadrant, followed by Tukey post-hoc analysis, while one-way ANOVA was used as a post-hoc slice test followed by Tukey analysis for multiple comparisons across treatments. Asterisks on connecting lines represent significant differences across conditions (including control and vehicle). (*), (**), and (***) correspond to *p* < 0.05, 0.01, and 0.001, respectively.

However, in HT-29, CIS induced both late-stage apoptosis (CIS × time, *p*-value = 0.006, F (2, 30) = 5.986, ηp^2^ = 0.285) and necrosis (CIS × time, *p*-value < 0.001, F (2, 30) = 10.407, ηp^2^ = 0.41) reaching 9.16 and 16.55% by 72 h for apoptosis and necrosis, respectively. However, as early as 24 h, the EO:CIS combination induced a slightly higher late apoptotic effect than CIS alone, albeit not significantly (EO × CIS, *p*-value > 0.05) (Figure 4). The representative flow cytometry dot plots are presented as Supplementary Material (Figure S4).

### 2.6. Effect on Colony Biomass of H460 and HT-29

**Figure 5:**
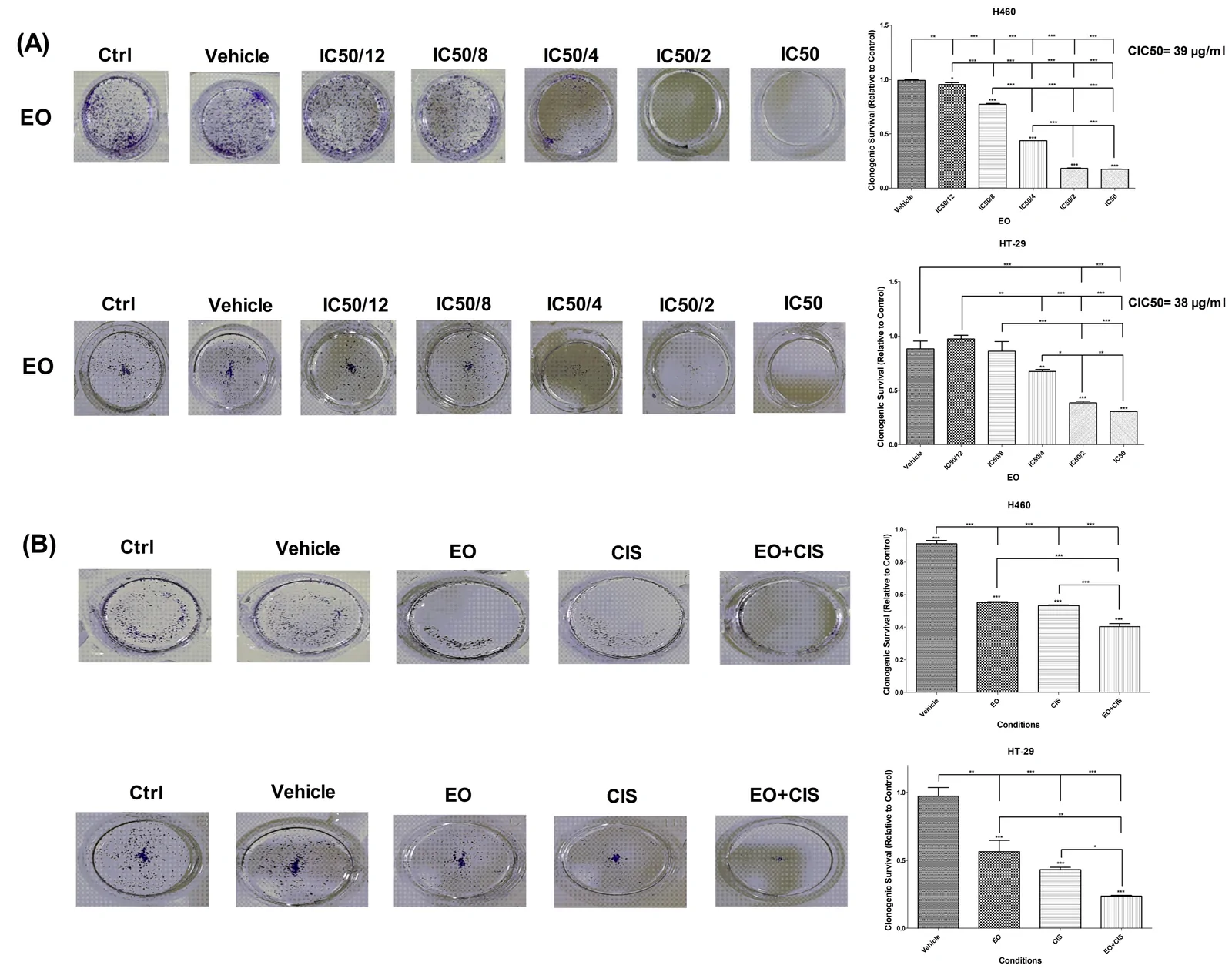
Representative data of crystal violet staining for the determination of colony biomass of H460 and HT-29. Images were captured under a low-power macro-magnification of 1.5×. Data analysis for CIC_50_ determination (Concentration inhibiting 50% of colony biomass) was conducted. The effect of EO, CIS and combination (CIC_50_:CIC_50_) on colony biomass was determined and quantified via absorbance measurement at 571 nm using an ELISA/microplate reader. Data presented is the mean ± SD from three different experiments. Statistical significance was performed using one-way ANOVA followed by Tukey post-hoc analysis for multiple dose comparison and two-way ANOVA for interaction dynamics evaluation between EO and CIS. Asterisks on bars represent significance relative to the control. Asterisks on connecting lines represent significant differences across groups (including DMSO vehicle group). (*), (**), and (***) correspond to *p* < 0.05, 0.01, and 0.001, respectively.

According to Figure 5A and Supplementary Figure S5, treatments with EO and CIS induced a concentration-dependent effect on colony biomass in both cell lines. The CIC_50_ (concentration inhibition 50% of colony biomass) was determined. The CIC_50_ values of EO and CIS were found to be 39 µg/mL and 0.55 µg/mL, respectively, in H460, whereas, in HT-29, the CIC_50_ values were found to be 38 µg/mL for EO and 0.95 µg/mL for CIS.

Furthermore, the effect of combination of both treatments on colony biomass in both cell lines was tested via their respective CIC_50_. Interestingly, the combination EO:CIS was found to have a higher effect on colony biomass compared to each experimental product alone for H460 (EO × CIS, *p*-value < 0.001, F (1, 40) = 178.847, ηp^2^ = 0.817) and HT-29 (EO × CIS, *p*-value < 0.001, F (1, 10) = 6.668, ηp^2^ = 0.4) (Figure 5B).

### 2.7. Effect on Migration of H460 and HT-29

**Figure 6:**
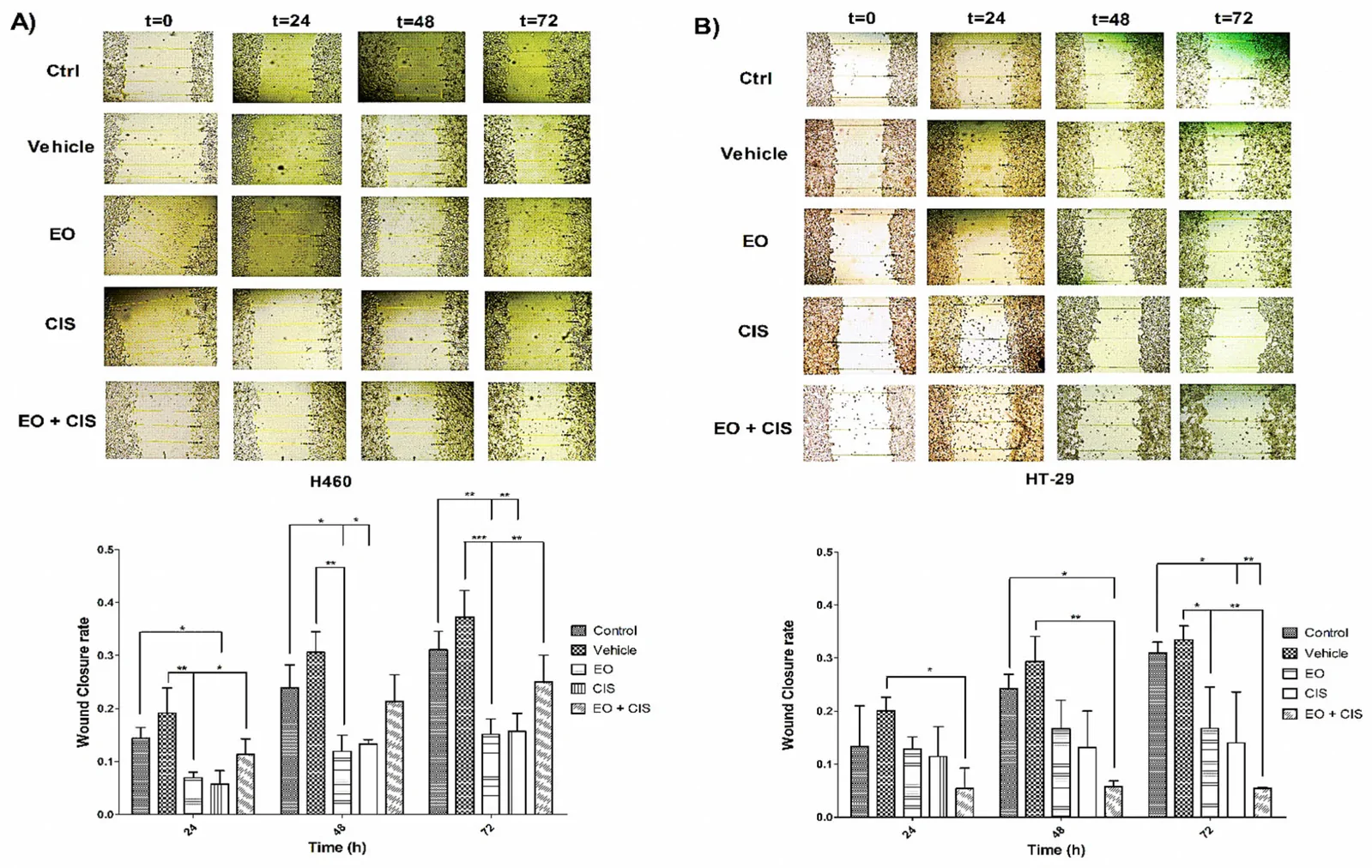
Effect of EO and CIS combination on the wound closure rate of H460 (**A**) and HT-29 (**B**) at 24 h, 48 h and 72 h as determined by the wound healing assay. Representative images and corresponding wound width measurements were captured at 0, 24, 48 and 72 h under 5× magnification. Data presented is the mean ± SD from three different experiments. Statistical significance was performed via two-way ANOVA (EO × CIS) and three-way ANOVA (Time × EO × CIS) for evaluating interaction dynamics within and across time points. Asterisks on connecting lines represent significant differences across experimental groups (including the untreated control and the DMSO vehicle groups). (*), (**), and (***) correspond to *p* < 0.05, 0.01, and 0.001, respectively.

In H460, EO and CIS induced similar wound closure of up to 15% at 72 h in comparison to untreated and DMSO vehicle group where migration reached nearly 26 and 37%, respectively. However, a lower effect was significantly observed with the combinatorial treatment compared to each individual treatment across all time points (EO × CIS, *p*-value < 0.001, F (1, 30) = 65.709, ηp^2^ = 0.687). The opposite phenomenon is observed with HT-29 where, even though each individual product showed a reduction in wound closure, a more pronounced effect was observed with the combination at the various time intervals, albeit non-significantly (EO × CIS, *p*-value > 0.05) (Figure 6).

### 2.8. Effect on Nuclear Morphology of H460 and HT-29

**Figure 7:**
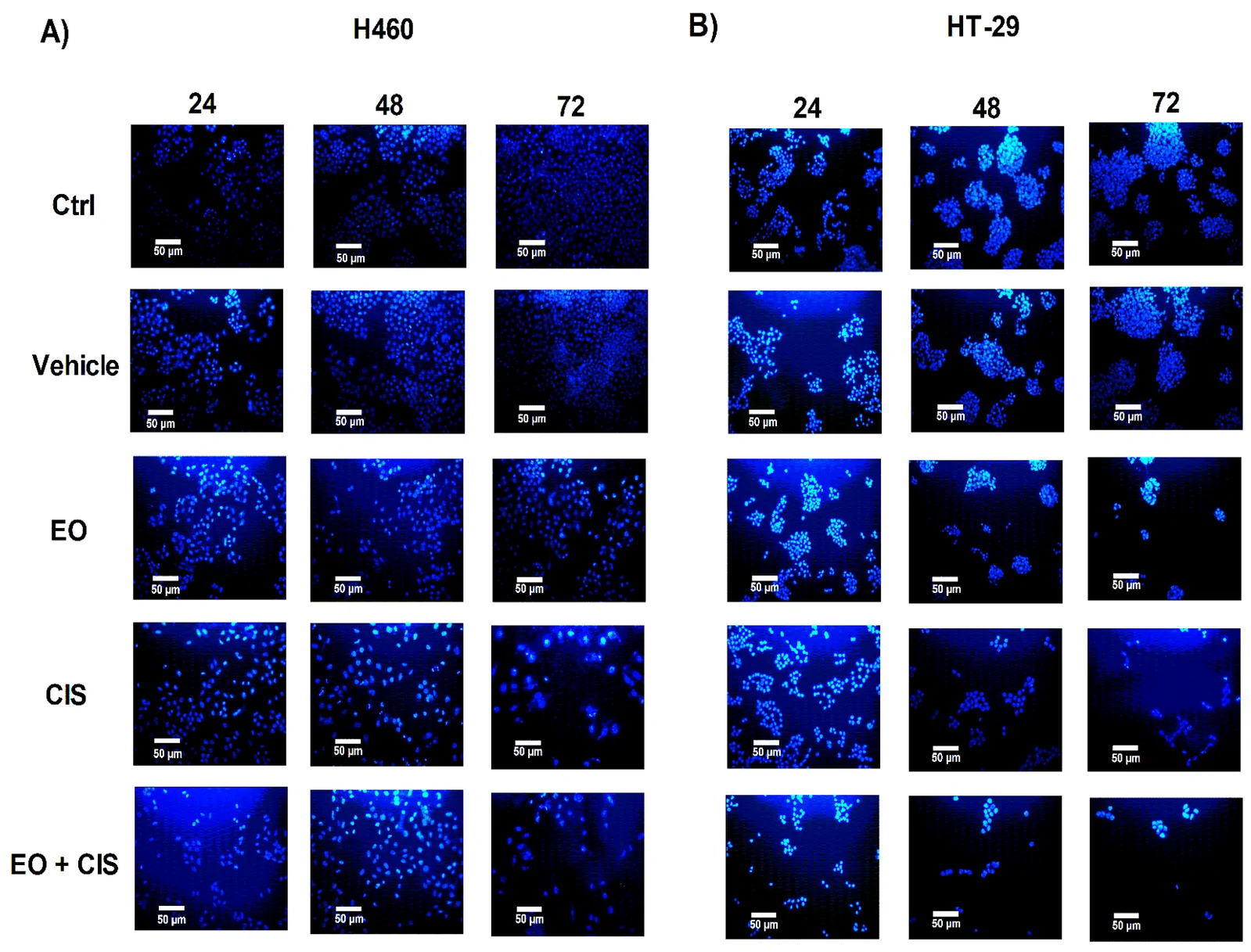
Effect of EO, CIS and their combination on the morphology of (**A**) H460 and (**B**) HT-29 at 24, 48 and 72 h via DAPI staining, detected via fluorescent microscopy. Scale bars represent 50 µm.

In H460 and HT-29, a reduction in the number of stained nuclei was predominantly shown in response to EO at 48 and 72 h, with some slight alteration in cell morphology observed. However, more visible alteration was observed in response to the CIS treatment, as particularly shown at 72 h. The EO:CIS combination, on the other hand, was found to predominantly induce a higher visible reduction in the number of stained nuclei in both cell lines, particularly at 24 h, with no significant increase in morphological alterations compared to each individual treatment (Figure 7).

### 2.9. Effect of EO:CIS Combination on NBT Reduction in H460 and HT-29

**Figure 8:**
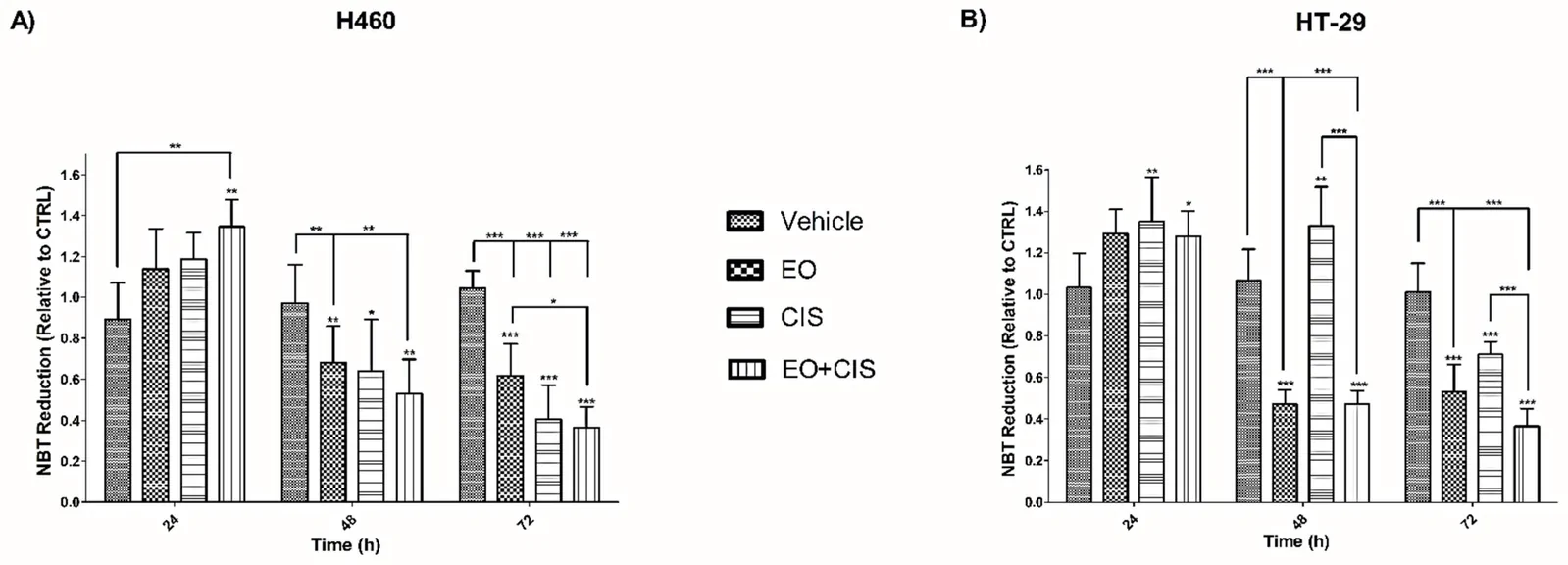
Effect of EO, CIS and their combination on NBT Reduction of H460 (**A**) and HT-29 (**B**) at 24, 48 and 72 h as determined via an NBT reduction assay. Data presented is the mean ± SD from three different experiments. Statistical significance was performed using two-way/three-way ANOVA for evaluating interaction dynamics within/across time points. Asterisks on bars represent significance relative to control. Asterisks on connecting lines represent significant differences across conditions (including vehicle). (*), (**), and (***) correspond to *p* < 0.05, 0.01, and 0.001, respectively.

In H460 cell lines, EO and CIS induce significant NBT reduction at 24 h. Although the combination exhibits a mildly more pronounced effect at 24 h, this difference did not reach statistical significance (EO × CIS, *p* > 0.05). Similarly, the initial increase was also observed in HT-29 cell line at this said time point in response to EO and CIS, although the combination did not show an increase compared to either treatment alone. In contrast, extended incubation at 48 and 72 h revealed a distinct metabolic shift as evidenced by the marked decrease in NBT reduction below control levels, with the combination showing a slightly more pronounced decrease at 48 h in H460 and 72 h in HT-29 cell lines (Figure 8).

## 3. Discussion

Previous studies have shown that essential oil components from cedrus species exhibit anti-cancer effects [6,11]. C. *libani*-derived wood oil has shown potency and chemo-enhancing effects against several cancer cell lines [10,12,13,14]. On the other hand, leaf extracts of *C. libani* have shown antioxidant, anti-inflammatory and anti-microbial activities, with no documented anticancer studies to date [8,16,17,18]. The current study investigates the leaf-derived EO, individually and combined with CIS, against H460 and HT-29 cell lines. The current EO yield and its chemical analysis differed from previous studies, potentially due to chemical polymorphism, geographic location, altitude, soil conditions, age of the tree, environmental stressors and ecotype/chemotype variability [19,20]. In fact, the current EO yield (0.045% *w*/*w*) is lower than previously reported (0.21% *w*/*w*), consistent with leaves yielding less oil than cones (0.41% *w*/*w*) and wood (0.68% *w*/*w*) [15]. Caryophyllene oxide is the most predominant component present within the studied EO alongside ß-caryophyllene and minor components such as germacrene D, contrasting with the literature reporting germacrene D and ß-caryophyllene as main markers of *C. libani* leaves cultivated in the Hadath eljubi-Tanourine Reserve and α/ß-pinenes as predominant in *C. libani* cultivated in Turkey [15,21].

In terms of anti-proliferative activity, HT-29 showed better responsiveness to the EO compared to H460, yet higher resistance to CIS, consistent with CIS’s known lower efficiency towards colorectal cancers (CRC), a fact attributed to the slower CRC growth, TP53 mutational status and enhanced resistance [22]. Interestingly, the lower overall potency of EO compared to CIS may reflect the complexity of the mixture, where constituents’ interactions result in synergy or antagonism [23]. Notably, studies on cedar wood oil demonstrated activity comparable to CIS and not significantly different from its isolated compounds (himachalene and himachalol), whereas the comparatively weaker effect of the leaf EO may stem from its broader chemical composition that dilutes the active constituents or introduces antagonistic interactions [10,12,13,14].

Different EO:CIS combination ratios based on IC_50_, IC_25_ and IC_12.5_ at 72 h were tested across various time points using 3 × 3 factorial combination matrices. While the three-way interaction (time × EO × CIS) was not significant in both H460 and HT-29 cell lines, time-dependent evaluation revealed that EO:CIS interaction is a dynamic shifting process rather than a static one. In the H460 cell line, three-way ANOVA confirmed significant main effects and EO × CIS interaction, particularly at 24 h and in response to the IC_12.5_ value of EO, whereas IC_25_ and IC_50_ maintained, respectively, a consistent antagonistic and additive profile across time points. Notably, in HT-29, a powerful acute synergistic window emerged 24 h before transitioning towards an additive response. These chemo-modulatory effects align with broader pharmacological evidence; for instance, related sesquiterpenoid constituents, such as himachalol, have been shown to exhibit synergistic interactions with CIS on various cancer cell lines, though context-dependent variations, such antagonism in melanoma, may suggest cell-dependent chemo-modulating effects. For subsequent downstream analysis, the IC_50_:IC_50_ ratio was selected to evaluate the time-dependent impact of individual treatments and their combination [12,13].

A trypan blue exclusion assay complemented MTT by directly quantifying viable and dead cells. Individual EO and CIS time-dependently reduced the number of viable cells in H460 and HT-29. At 72 h, EO treatment decreased viability to ~50% of control, consistent with MTT findings. Interestingly, CIS exhibited greater reduction in viability by trypan blue compared to MTT, since enhanced mitochondrial activity in stressed cells overestimates viability in MTT-based assays [24]. The combination produced greater growth inhibition than either treatment alone, as early as 24 h. EO alone did not induce significant cell death. In HT-29 cells, the combination induced moderately greater cell death than CIS alone, supporting a chemo-enhancing role for EO. In H460 cells, comparable cell death between CIS and the combination at 72 h suggest that early growth inhibition at 24 h may reflect transient proliferative suppression rather than increased cytotoxicity.

Annexin V/Propidium Iodide further characterized the mode of cell death. EO showed no detectable cytotoxicity through 72 h, reinforcing our trypan blue findings. In H460, CIS induced necrosis starting 48 h, whereas the combination reduced necrotic death, suggesting a transient protective interaction. However, it converges to CIS necrosis levels at 72 h. In HT-29, CIS and the combination produced comparable necrotic and late-apoptotic cell death. The predominance of necrosis, despite CIS’s reported apoptotic mechanism, may reflect excessive cellular stress at the doses used compared to the suboptimal doses adopted in previous studies [25,26]. The higher cytotoxicity detected by trypan blue, compared to Annexin V/PI, may reflect early membrane permeability preceding classical death markers. However, more mechanistic investigations are warranted to elucidate the underlying factors contributing to this discrepancy.

A long-term biomass assay assessed colony biomass formation. Both EO and CIS showed dose-dependent reduction in colony biomass in H460 and HT-29, which is consistent with the long-term inhibitory effects of CIS, as previously reported, and representing the first clonogenic report of a *C. libani* extract [27]. CIC_50_ values were significantly lower than MTT IC_50_ values, reflecting the greater sensitivity of long-term biomass evaluation over an extended period. Additionally, EO:CIS co-treatment produced greater inhibition in both cell lines compared to each individual CIC_50_ value. These data suggest enhanced inhibition under our condition but do not confirm a definitive reversal of cisplatin resistance.

Since metastasis is the leading cause of cancer-related death, inhibiting cancer cell migration is of therapeutic importance [28]. EO and CIS individually demonstrated comparable reduction in wound closure in H460 and HT-29 over 72 h, consistent with CIS’s known anti-proliferative and anti-migratory activity according to previous reports [29,30]. However, the EO:CIS combination showed opposite responses between the two lines; enhanced closure reduction in HT-29 but a lesser effect in H460, reflecting differences in molecular contexts and baseline migratory capacity. HT-29, with its higher epithelial phenotype, may exhibit lower wound closing ability, rendering it more prone to the combined treatment effect, whereas H460 may activate compensatory pathways conferring resistance, though more mechanistic studies are still required to confirm such hypothesis [31,32]. Nonetheless, the use of IC_50_ values in our wound healing experiments makes it difficult to separate anti-proliferative or cytotoxic effects from a true anti-migratory effect, particularly in later time points. While our observations point to a potential influence of our treatments on cellular behavior and movement, definitive conclusions regarding anti-migratory mechanisms warrant further investigations using sub-cytotoxic doses such as IC_25_ or IC_12.5_ or targeted molecular studies.

DAPI staining evaluates nuclear morphology, which can reflect characteristics associated with nuclear fragmentation and cytotoxicity. EO-derived components from *Cedrus* species, particularly *C. atlantica* and several anti-neoplastic agents including CIS, have demonstrated strong cytotoxic effects [33,34,35]. In our study, EO alone induced a time-dependent reduction in stained nuclei with mild morphological alterations. CIS, by contrast, induced more pronounced nuclear alterations, particularly at 72 h, with stronger effects in H460 than in HT-29, in line with the trypan blue cytotoxicity data. Although the combination mildly increased nuclear alterations at 72 h, its more prominent effect is the reduction in stained nuclei, as early as 24 h, compared to either treatment alone, corroborating the earliest and greatest growth inhibition, as previously observed. However, more research is still needed to provide a more comprehensive quantitative evaluation.

Although oxidative stress plays a dual role, DNA-damaging agents such as CIS generate ROS as part of their genotoxic mechanisms [36]. *C. atlantica* extracts and *C. libani* tar similarly demonstrated anti-cancer activity via ROS generation and DNA damage [34,35,37]. An NBT reduction assay served in our study as a marker of intracellular superoxide generation and metabolic activity in H460 and HT-29. At 24 h, CIS exhibited an upregulation in NBT reduction, which aligns with its known cytotoxic–genotoxic profile. A similar pattern was observed following EO treatment with a mild elevation with the combination group compared to either individual treatment, which suggests an acute surge in intracellular redox level. In contrast, HT-29 displayed similar early alterations with single agents, where the combination group showed no increase in NBT reduction compared to either treatment alone, potentially pointing to distinct stress handling mechanisms between both cell lines. As incubation duration extends over 48 and 72 h, a progressive overall decline in NBT reduction was observed across both cell lines. While this decrease reflects a reduction in superoxide-reducing capacity, it is consistent with the cumulative loss of cell viability and overall metabolic activity over time.

## 4. Materials and Methods

### 4.1. Harvesting and Extraction

Approximately 1 kg of fresh leaves from *Cedrus libani* A. Rich. were collected during the spring season of 2024 from wild growing trees in Laqlouq, Mount Lebanon Governorate, Lebanon (34°08′ N, 35°52′ E, approximatively 1700 m altitude). While a physical voucher specimen was not deposited, the plant material was confirmed by Dr. Antoine Saab, professor of biochemistry and biotechnology at the Lebanese University, Faculty of Sciences, who has previously investigated the biological properties of *Cedrus libani*. Species identification was based on morphological characteristics and cross-referenced with the NCBI taxonomy database (Taxonomy ID: 93692).

After air drying under shade for two weeks (yielding approximatively 450 g of dry plant material), the essential oil (EO) was extracted at a ratio of 1:10 in distilled water by hydro-distillation using a Clevenger apparatus for 4 h, generating a yield of ~0.045% (weight of EO/dry plant weight). The obtained EO had a pale yellow to golden hue coloration with a strong spicy aroma and exhibiting a density of 0.89 g/mL. The oil was recuperated in a small glass amber vial maintained at 4 °C protected from light and humidity. To prepare the required experimental concentrations, pure EO was initially dissolved in DMSO (Fisher Chemical, Loughborough, UK) at a ratio of 1:1 prior to any subsequent dilutions. DMSO at a final concentration of ≤0.1% was used as vehicle control.

### 4.2. Gas-Chromatography–Mass Spectrometry (GC-MS)

GC-MS analysis was conducted using a Shimadzu QP2020 system coupled with a Nexis GC2030 gas chromatograph and an Agilent DB-5MS column (30 m, 0.25 mm × 0.25 μm film thickness) using a helium gas flow rate of 1.3 mL/min. The oven temperature was programmed as follows: 60 °C (5 min), increased to 220 °C at 4 °C/min, then to 280 °C at 11 °C/min, held for 15 min. The sample was diluted 100× in methylene chloride, and 1 μL was injected into split mode (1:50) at 280 °C. The MS interface and ion source were maintained at 280 °C and 230 °C, respectively, with a scan range of m/z 50–400. Compounds identification was performed using spectral matching against the NIST14 library. Match scores were not provided in the dataset, and no authentic standards or retention index data were available, therefore, identifications remain putative. GC-MS analysis was performed in triplicate technical injections of a single extraction batch to ensure analytical reproducibility.

### 4.3. Cell Lines and Culture

NSCLC H460 was cultured in RPMI 1640 medium (Corning, Manassas, VA, USA) and Colorectal Adenocarcinoma HT-29 in Dulbecco’s Modified Eagle Medium (DMEM) (Thermo Fisher Scientific, Waltham, MA, USA) supplemented with 10% fetal bovine serum (FBS) (Corning, Woodland, CA, USA) and 1% of streptomycin–penicillin–amphotericin B (Corning, Manassas, VA, USA). Cell lines were incubated in a humidified atmosphere of 5% CO_2_ at 37 °C.

The cells were a gift from Dr. Salem Chouaib (Gulf Medical University). H460 were purchased from AddexBio technologies (San Diego, CA, USA) (catalogue number C0016003), while the HT29 were purchased from Cytion GmBH (Heidelberg, Germany) (catalogue number 300215). All experiments were conducted at low passage numbers (8–12 passages) to reduce the phenotypic drift. All cells were tested for mycoplasma after thawing using a PCR-based mycoplasma detection kit and DAPI staining.

### 4.4. Cell Viability (MTT) Assay

The effect of each of EO and CIS (Cat. No. 15663-27-1, Thermoscientific chemicals, Acros Organic, Geel, Belgium) on the viability of H460 and HT-29 was determined by MTT (Cat. No. M5655-1G, Sigma-Aldrich, St. Luis, MO, USA), as described previously [38]. The cells were seeded in 96 well plates at densities optimized for each model (H460:80,000 cells/mL; HT-29:120,000 cells/mL). MTT was performed following 24, 48 and 72 h treatment periods. Briefly, at each time point, media was aspirated and fresh media (containing MTT reagent at 500 µg/mL) was added to each well and incubated for 2–3 h at 37 °C. The resulting purple formazan crystals were fully solubilized using DMSO and absorbance was measured at 595 nm. Blank correction was performed using a cell free EO–MTT blank. Cell viability was expressed as the ratio of proliferation in treated conditions compared with that of the untreated group. The inhibitory concentrations (IC_12.5_; IC_25_ and IC_50_) were determined using GraphPad Prism 5.00 by fitting % of cell response versus Log[concentration].

### 4.5. Drug Combination Treatment

Cells were treated with EO and/or CIS using the IC values obtained at 72 h. After treatment for 24, 48 and 72 h, cell viability was determined by an MTT assay. The effect of the various combinations between EO and CIS was determined from the single-agent effects measured at the exact same corresponding time point based on the Jin’s formula:

Q = E(A + B)/(EA + EB − EA × EB)

where E(A + B) is the inhibition rate (IR) of the combination of drug A and drug B; EA is the IR of drug A; and EB is the IR of drug B. A “Q” value ≤ 0.85 imply an antagonistic effect, where a Q value ranging between 0.85 and 1.15 implies an additive effect and a Q value ≥ 1.15 means a synergistic effect is obtained.

### 4.6. Trypan Blue Exclusion Assay

The viability/death of H460 and HT-29 were also determined using trypan blue (Cat. No. T8154, Sigma-Aldrich, St. Luis, MO, USA). Cells were treated for 24, 48, and 72 h using EO, CIS, and a combination at their corresponding IC_50_ values (72 h). The ratio of viability was measured by computing the number of viable cells in treated conditions to that of the untreated. On the other hand, the ratio of death in each condition (treated and untreated) was determined by computing the number of dead cells to the total number of cells (viable + dead) within each sample.

### 4.7. Annexin V/Propidium Iodide (PI) Staining Assay

An Annexin V Apoptosis Detection Kit with propidium iodide (PI) was used to detect apoptotic/necrotic cell death. Briefly, cells were plated at a density of 20 × 10^4^ cells in 35 mm Petri dishes. Following treatment for 24, 48, and 72 h with EO, CIS or their combination, the cells were collected at the indicated time points by trypsinization and subsequently washed with 1× PBS prior to labelling with Annexin V-APC and PI, following the manufacturer’s protocol (Kit Elabscience Cat #E-CK-A211). Acquisitions of 10,000 cells were performed using a BioRad S3E Cell Sorter and data processed using the FCS Express flow cytometry program (De Novo Software, Pasadena, CA, USA). Compensation was established using unstained controls for Annexin V-APC and PI. Gating was performed by first isolating the cellular population on FSC vs. SSC plots, excluding debris, and subsequently analyzing fluorescence intensity across four quadrants, representing viable (An−/PI−), early apoptotic (An+/PI−), late apoptotic (An+/PI+) and necrotic cells (Ann−/PI+). The data are determined as mean ± SD from three independent experiments (n = 3).

### 4.8. Long-Term Biomass Proliferation Assay via Crystal Violet Staining

The general protocol for the colony biomass determination assay was performed following the “plating before treatment” setup according to ref. [34,39]. Briefly, cells were seeded in 24 well plates (H460:2000 cells/mL; HT-29:6000 cells/mL) and treated with various fractions of the 72-h-derived IC_50_ of either EO or CIS (IC_50_, IC_50_/2, IC_50_/4, IC_50_/8 and IC_50_/12). After 8 days of incubation, cells were fixed and stained. GraphPad Prism 5.00 was used to fit the % response to determine the CIC_50_ of each treatment as:

% Response = f(log[conc.])

And the CIC_50_ is:

CIC_50_ = concentration inhibiting 50% of colony biomass.

The same experimental procedure was performed using the CIC_50_ of EO, CIS and combination to determine the effect of the combinatorial regimen on the colony biomass formation.

### 4.9. Wound Healing Assay for Cell Migration

Cells were seeded in 24 well plates and incubated until 90–100% confluency. Mitomycin C (Cat. No. 50-07-07, Sigma-Aldrich, Taufkirchen, Germany) was added into each well at a final concentration of 5 µg/mL for 2 h to arrest proliferation. A scratch was then made using a 100 µL tip and wells were washed with phosphate buffered saline (PBS) 1× (Corning, Manassas, VA, USA) three times to remove residues. Following treatment with EO, CIS or their combination at their corresponding IC_50_ values (72 h), representative pictures of the wounds including wound width measurements were taken at 0, 24, 48 and 72 h using phase contrast imaging. Rate of closure was determined by computing the wound width at each time point to that of the reference point (t_0_).

### 4.10. Nucleic Acid Staining Using DAPI

Sterile coverslips were coated onto 24 well plates prior to cell seeding. After seeding (H460:80,000 cells/mL; HT-29:100,000 cells/mL), cells were treated for 24, 48 and 72 h with EO, CIS, or their combination. Following fixation using 4% paraformaldehyde (PFA) (Sigma-Aldrich, Taufkirchen, Germany) and permeabilization with 0.1% Triton X-100 (Fisher Scientific, Geel, Belgium), cells were stained with DAPI Anti-Fade Mounting Medium (Cat. No. 637877, Sigma-Aldrich, Taufkirchen, Germany) and transferred onto slides for fluorescent microscopic detection. Scale bars were generated using ImageJ based on the spatial calibration of the microscopy images.

### 4.11. NBT Reduction Assay

Cells were seeded in 96 well plates (H460:80,000 cells/mL; HT-29:120,000 cells/mL) then treated for 24, 48 and 72 h with EO, CIS or their combination. The level of ROS generated was determined using the nitro-blue tetrazolium (NBT) assay [40]. In brief, NBT (Cat. No. 415925000, Acroorganics, Geel, Belgium) was added to each well (1000 µg/mL, 100 μL) and incubated for 1 h. Cells were then washed with methanol and the formed formazan crystals were solubilized with KOH (2M, 120 μL–Sigma-Aldrich, St. Louis, MO, USA) and DMSO (140 μL). The absorbance of the developed color was read at 620 nm using a Multiskan™ FC ELISA/microplate reader (Thermo Scientific, Waltham, MA, USA).

The rate of reduced NBT was determined as follows:

Rate of NBT reduction not % = (Abs of treated cells/Abs of untreated)

### 4.12. Statistical Analysis and Experimental Design

All experiments were performed at least in triplicates (n = 3) as three independent biological experiments performed separately, and the data are presented as mean ± standard deviation (SD). For IC_50_, IC_25_ and IC_12.5_ determination, % response versus log-concentration (% Response = f(log[conc.])) curves were plotted using GraphPad Prism 5.00. Statistical significance was determined by SPSS version 22 using multi-factorial ANOVA followed by Tukey post-hoc analysis. For single-agent assays using various concentrations of EO or CIS across time points, a three-way ANOVA was performed (cell × time × conc.). Similarly, a three-way ANOVA (time × EO × CIS) was employed for the comprehensive drug combination study using IC_50_, IC_25_ and IC_12.5_ concentrations to evaluate the overall main effect and interaction kinetics across all combination ratios and time-points. For time-dependent evaluations using fixed IC_50_ concentrations, a two-way ANOVA (EO × CIS) and three-way ANOVA (time × EO × CIS) were performed. Across all experimental models, a one-way ANOVA was utilized as a post-hoc slice test to examine simple main effects. GraphPad Prism 5.00 was used to generate all graphs, and Microsoft Excel was used to construct the 3D figure for the 3 × 3 factorial design.

EO was compared against both untreated and DMSO vehicle groups. Single-agent CIS assays were evaluated against untreated and vehicle aqueous controls, whereas subsequent combination and fixed-concentration experiments compared CIS-treated groups against both untreated controls and their corresponding combination treatments.

## 5. Conclusions

This study provides in-vitro preliminary evidence for the anti-proliferative effect of extracts derived from *C. libani* and their context-dependent interaction with cisplatin. Further investigations are required to obtain a more comprehensive cytotoxic profile regarding mechanistic pathways, chemo-modulation and selectivity against only cancer cells versus normal ones.

## Figures and Tables

**Table 1:** EO compounds putatively identified via GC-MS.

Retention Time (min, Mean ± SD)	Compound	Relative Area (%, Mean ± SD)
6.4 ± 0.001	α-Pinene (MH)	8.44 ± 0.15
6.98 ± 0.002	Camphene (MH)	0.86 ± 0.02
7.84 ± 0.0005	Sabinene (MH)	0.18 ± 0.02
8.01 ± 0.002	ß-Pinene (MH)	1.56 ± 0.01
8.49 ± 0.002	ß-Myrcene (MH)	9.63 ± 0.21
9.22 ± 0.002	3-Carene (MH)	0.53 ± 0.04
9.87 ± 0.002	O-Cymene (MH)	0.5 ± 0.03
10.06 ± 0.002	D-Limonene (MH)	7.46 ± 0.09
13.19 ± 0.003	Nonanal (Other)	0.53 ± 0.06
14 ± 0.003	α-Campholenal (OM)	0.41 ± 0.04
14.76 ± 0.002	Camphor (OM)	0.29 ± 0.03
16.14 ± 0.003	Terpinene-4-ol (OM)	1.73 ± 0.1
16.69 ± 0.005	(1R)-(-)-Myrtenal (OM)	0.42 ± 0.03
17.13 ± 0.001	Verbenone (OM)	0.43 ± 0.06
18.07 ± 0.007	Anisole, 2-Isopropyl-5-Methyl-(Other)	0.27 ± 0.002
20.11 ± 0.002	Bornyl acetate (OM)	13.8 ± 0.23
20.53 ± 0.002	Trans-pinocarvyl acetate (OM)	0.24 ± 0.08
21.52 ± 0.002	Myrtenyl acetate (OM)	0.44 ± 0.04
22.83 ± 0.007	Neryl acetate (OM)	0.34 ± 0.04
23.4 ± 0.003	Copaene (SH)	0.81 ± 0.02
23.52 ± 0.003	Geranyl acetate (OM)	0.52 ± 0.02
23.68 ± 0.002	(-)-ß-Bourbonene (SH)	0.68 ± 0.04
23.89 ± 0.001	Germacrene D Form 1-tentative (SH)	0.32 ± 0.06
24.9 ± 0.001	ß-Caryophyllene (SH)	7.76 ± 0.02
25.25 ± 0.001	ß-Copaene (SH)	0.57 ± 0.04
26.11 ± 0.001	α-Caryophyllene (SH)	2.06 ± 0.07
26.76 ± 0.001	ß-Muurolene (SH)	3.3 ± 0.2
26.9 ± 0.005	Cyclosativene (SH)	1.37 ± 0.34
26.95 ± 0.001	Germacrene D Form 2- tentative (SH)	0.92 ± 0.24
27.54 ± 0.002	α-Muurolene (SH)	1.92 ± 0.01
28 ± 0.002	γ-Muurolene (SH)	1.49 ± 0.03
28.17 ± 0.003	Cadinene (SH)	0.76 ± 0.03
28.26 ± 0.005	2-(1-Cyclopent-1-Enyl-1-Methyl-Ethyl)-Cyclopentanone (Other)	0.57 ± 0.04
28.59 ± 0.002	Oxacyclotridecan-2-one (Other)	1.77 ± 0.03
28.75 ± 0.002	Naphthalene, 1,2,4a,5,6,8a-hexahydro-4,7-dimethyl-1-(1-methylethyl)- (Other)	0.4 ± 0.02
28.89 ± 0.004	Bisabolene (SH)	1.33 ± 0.16
29.2 ± 0.003	Trans-ß-Caryophyllene Oxide (OS)	0.87 ± 0.07
29.58 ± 0.05	α-Calacorene (SH)	0.28 ± 0.07
29.72 ± 0.003	(1S,3aR,4R,8R,8aS)-1-Isopropyl-3a-Methyl-7-Methylenedecahydro-4,8-Epoxyazulene (OS)	1.23 ± 0.28
29.86 ± 0.01	2-Hepten-1-one, 1-(2-Hydroxy-5-Methylphenyl)-(Other)	0.52 ± 0.29
30.16 ± 0.001	Caryophyllene Oxide (OS)	21.16 ± 0.21
30.27 ± 0.001	(1R,7S,E)-7-Isopropyl-4,10-Dimethylenecyclodec-5-enol (OS)	1.1 ± 0.06

Retention time (min) and relative area (%) are presented as the mean ± standard deviation (SD) of three analytical replicates (three distinct injections of a single extraction batch). Abbreviations: SD, standard deviation; MH, monoterpene hydrocarbon; SH, sesquiterpene hydrocarbon; OM, oxygenated monoterpene; and OS, oxygenated sesquiterpene.

**Table 2:** IC values (IC_50_, IC_25_ and IC_12.5_ in µg/mL) of EO on H460 and HT-29.

IC Values of EO (µg/mL)			
Time (h)	IC_50_	IC_25_	IC_12.5_
H460			
24	238 (216–273)	156 (138–174)	103 (ns–126)
48	200 (185–220)	136 (121–153)	92 (ns–114)
72	165 (152–178)	114 (100–127)	70 (ns–92)
HT-29			
24	207 (198–218)	142 (130–153)	94 (67–110)
48	173 (162–185)	110 (95–124)	57 (ns–85)
72	132 (121–142)	85 (75–95)	62 (54–71)

IC Values are determined via non-linear regression fit from three independent experiments (n = 3), plotting % Response = f(log[conc.]) via GraphPad Prism 5.00. Values represent the best-fit estimates with corresponding 95% confidence intervals (95% CI) shown in parentheses. Abbreviations: IC, inhibitory concentration and EO, essential oil.

**Table 3:** Q values of EO and CIS on H460 and HT-29.

H460			
Q 24/48/72 h			
CIS	IC_50_	IC_25_	IC_12.5_
EO			
IC_12.5_	1.65/0.95/0.84	1.26/1.08/1.1	1.22/1.00/1.11
IC_25_	0.71/0.74/0.68	0.58/0.77/0.89	0.7/0.83/1.00
IC_50_	1.05/1.19/1.16	1.09/1.15/1.1	0.98/1.07/1.07
**HT-29**			
Q 24/48/72 h			
CIS	IC_50_	IC_25_	IC_12.5_
EO			
IC_12.5_	2.27/0.78/1.02	2.23/0.87/1.05	1.31/0.91/1.11
IC_25_	6.23/1.01/1.12	4.97/1.15/1.15	2.37/1.04/1.19
IC_50_	2.98/0.9/0.98	2.27/1.07/0.98	1.63/1.11/1.08

Q values were calculated for 3 × 3 combination matrix between EO and CIS across 24, 48 and 72 h. Data represents the mean Q values calculated using Jin’s formula from three independent experiments (*n* = 3), based on time-matched single-agent responses. Abbreviations: EO, essential oil; CIS, cisplatin; and IC, inhibitory concentration. Classification: Q < 0.85, antagonistic; 0.85 < Q < 1.15, additive; and Q > 1.15, synergy.

## Data Availability

The original contributions presented in this study are included in the article/supplementary material. Further inquiries can be directed to the corresponding author.

## Supplementary Materials

The following supporting information can be downloaded at: https://www.mdpi.com/article/10.3390/ijms27209044/s1.

## Abbreviations

CIS	cisplatin
CIC_50_	clonogenic inhibitory concentration (50%)
DMEM	Dulbecco’s Modified Eagle Medium
DMSO	dimethyl Sulfoxide
DAPI	4′,6-diamidino-2-phenylindole
EO	essential oil
FBS	Fetal Bovine Serum
GC-MS	gas chromatography–mass spectrometry
IR	inhibition rate
IC_50_	half-maximal inhibitory concentration (producing 50% inhibition)
IC_25_	inhibitory concentration (producing 25% inhibition)
IC_12.5_	inhibitory concentration (producing 12.5% inhibition)
KOH	potassium hydroxide
MTT	3-(4,5-dimethylthiazol-2-yl)-2,5-diphenyltetrazolium bromide
NSCLC	non-small cell lung cancer
NBT	nitro blue tetrazolium
PBS	phosphate-buffered saline
PI	propidium iodide
Q	interaction score
ROS	reactive oxygen species
RPMI	Roswell Park Memorial Institute
