# Supplementary material for: In Vitro Anti-Proliferative and Cisplatin-Modulating Effects of Cedrus libani A. Rich Leaf Essential Oil on H460 and HT-29 Human Cancer Cell Lines
Source: Int J Mol Sci. 2026 Oct 12;27(20):9044. doi: 10.3390/ijms27209044 (PMC13704914; doi:10.3390/ijms27209044)
Supplement: Supplementary file 1 [file ijms-27-09044-s001.zip › ijms-4596833-supplementary.pdf]

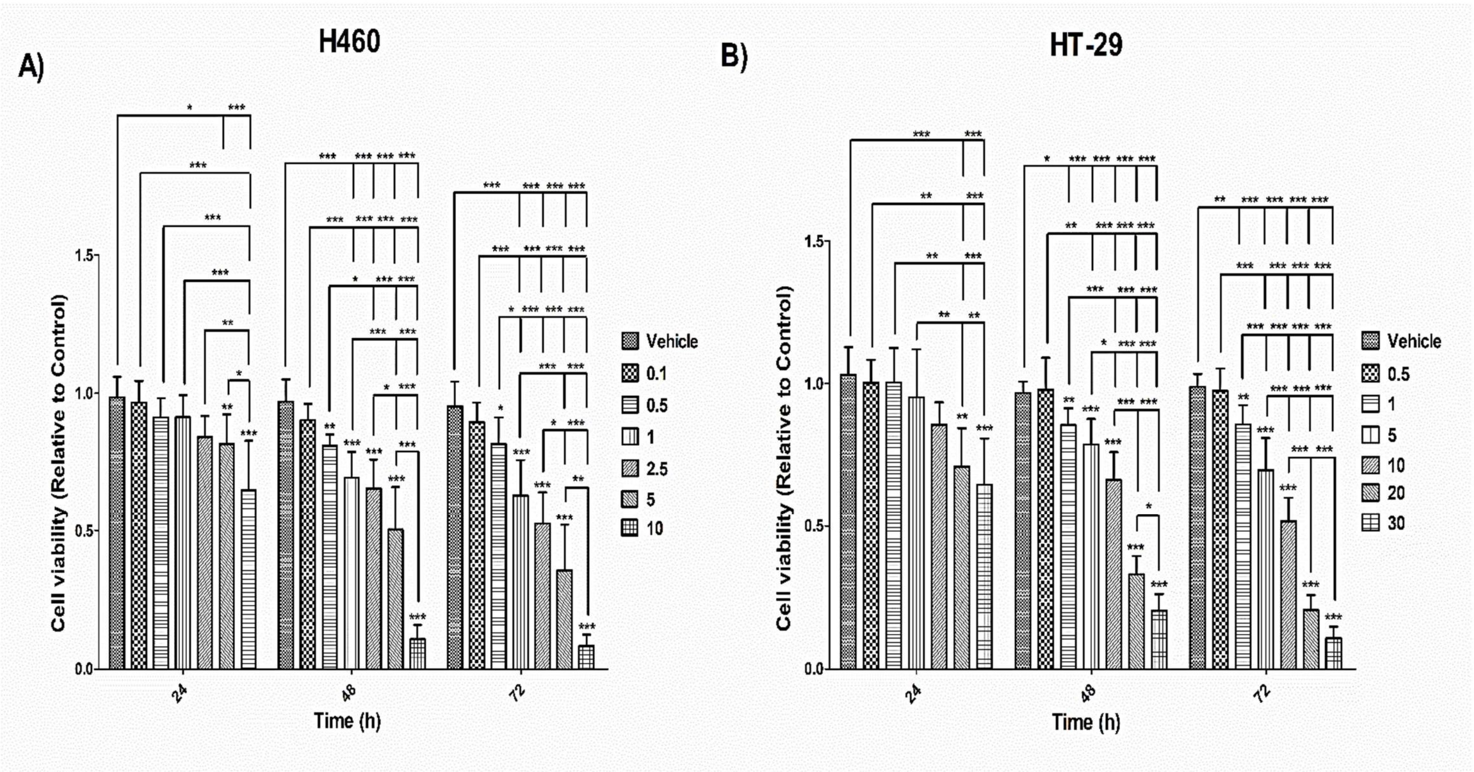

Figure S1. Effect of various concentrations (in µg/ml) of cisplatin (CIS) on cell viability of H460 (A) and HT-29 (B) at 24, 48 and 72h as determined via MTT assay. Data presented is the mean ± SD from three different experiments. Statistical evaluation was conducted using a hierarchical approach: a three-way ANOVA (Cell*Time*Conc.) and two-way ANOVAs (Time*Conc. within each cell line) evaluated global main effects and interactions, while one-way ANOVAs served as post-hoc slice tests for multi-dose comparisons within individual time points. Group means were compared using Tukey's multiple comparisons test. Asterisks on bars denote significance relative to control. Asterisks on connecting lines represent significant differences across specific treatment groups (including the DMSO vehicle group). No significant differences were observed between the untreated and vehicle controls. (*), (**), and (***) correspond to p< 0.05, 0.01, and 0.001, respectively.

Table S1: Time-specific IC values (IC_{50}, IC_{25} and $IC_{12.5}$ in µg/ml) of CIS on H460 and HT-29 determined using dose-response non-linear regression, plotting % Response = f(log[conc.]) via GraphPad Prism 5.00.

	**IC values of CIS (µg/ml)**		
**Time (h)**	IC_{50}	IC_{25}	$IC_{12.5}$
	**H460**		
24	13.7 (ns)	3.98 (3.17 – 4.8)	1.05 (0.7 – 1.53)
48	3.49 (2.91 – 4.13)	0.98 (0.717 – 1.28)	0.055 (ns)
72	1.87 (1.57 – 2.21)	0.56 (0.44 – 0.7)	0.15 (ns – 0.27)
	**HT-29**		
24	23.8 (21.77 – 26.3)	10.2 (8.01 – 12.6)	3.38 (ns – 5.34)
48	15.13 (13.5 – 17)	6.82 (5.22 – 8.41)	ns
72	10.1 (8.83 – 11.6)	3.58 (2.41 – 4.88)	0.79 (ns – 1.83)

IC Values are determined via non-linear regression fit from three independent experiments (n=3), plotting % Response = f(log[conc.]) via GraphPad Prism 5.00. Values represent the best-fit estimates with corresponding 95% confidence intervals (95% CI) shown in parentheses.
Abbreviations: IC, inhibitory concentration, CIS, cisplatin.

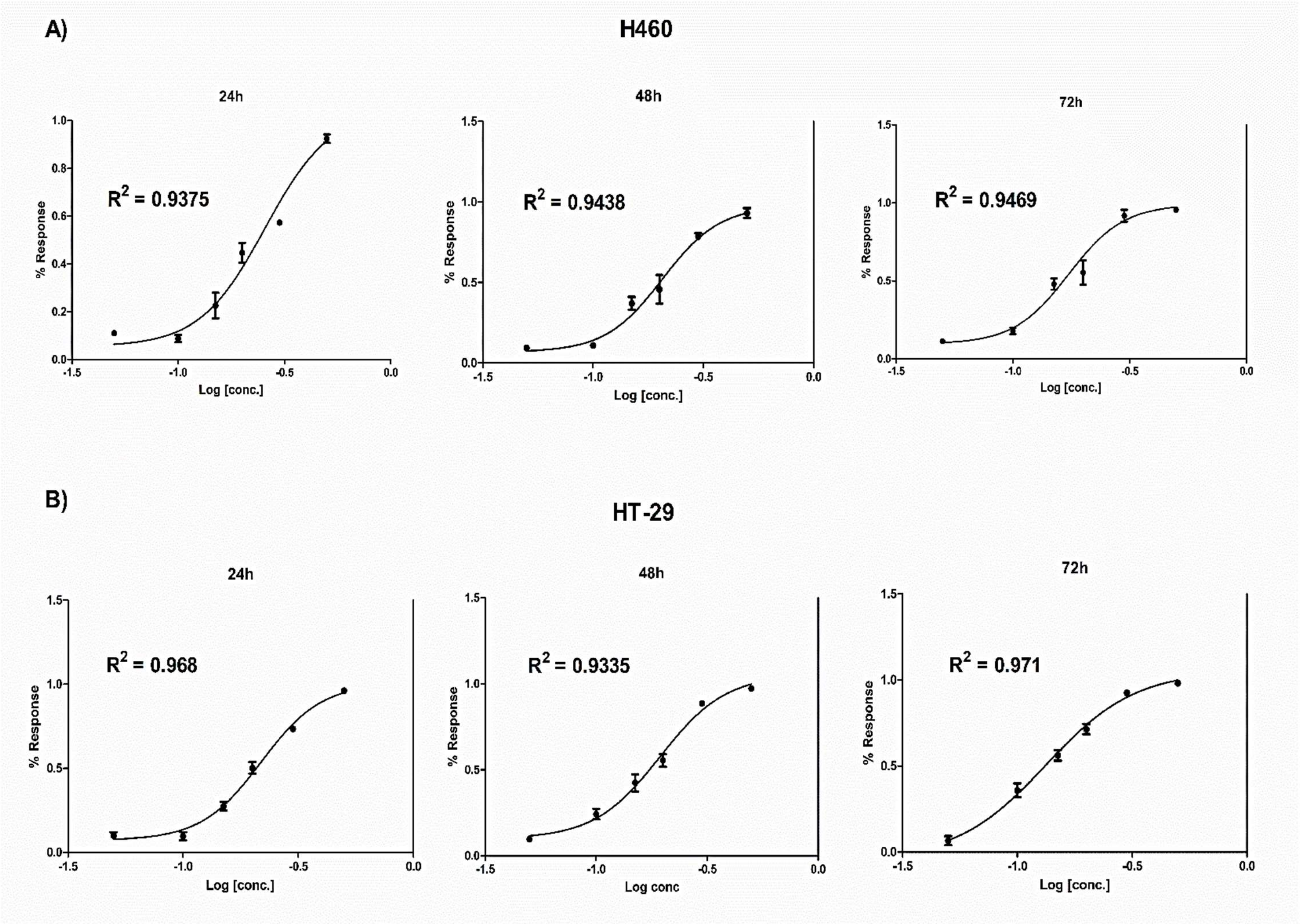

Figure S2. Non-linear regression dose-response curves for EO in H460 **(A)** and HT-29 **(B)** at 24, 48, and 72 h. Response ratio is plotted as a function of log- concentration. Curves represent non-linear fits generated using a variable-slope sigmoidal dose-response model. Data points are presented as the mean ± SD from three independent experiments (n = 3). Goodness-of-fit (R^2) values for each curve are indicated within the respective panels.

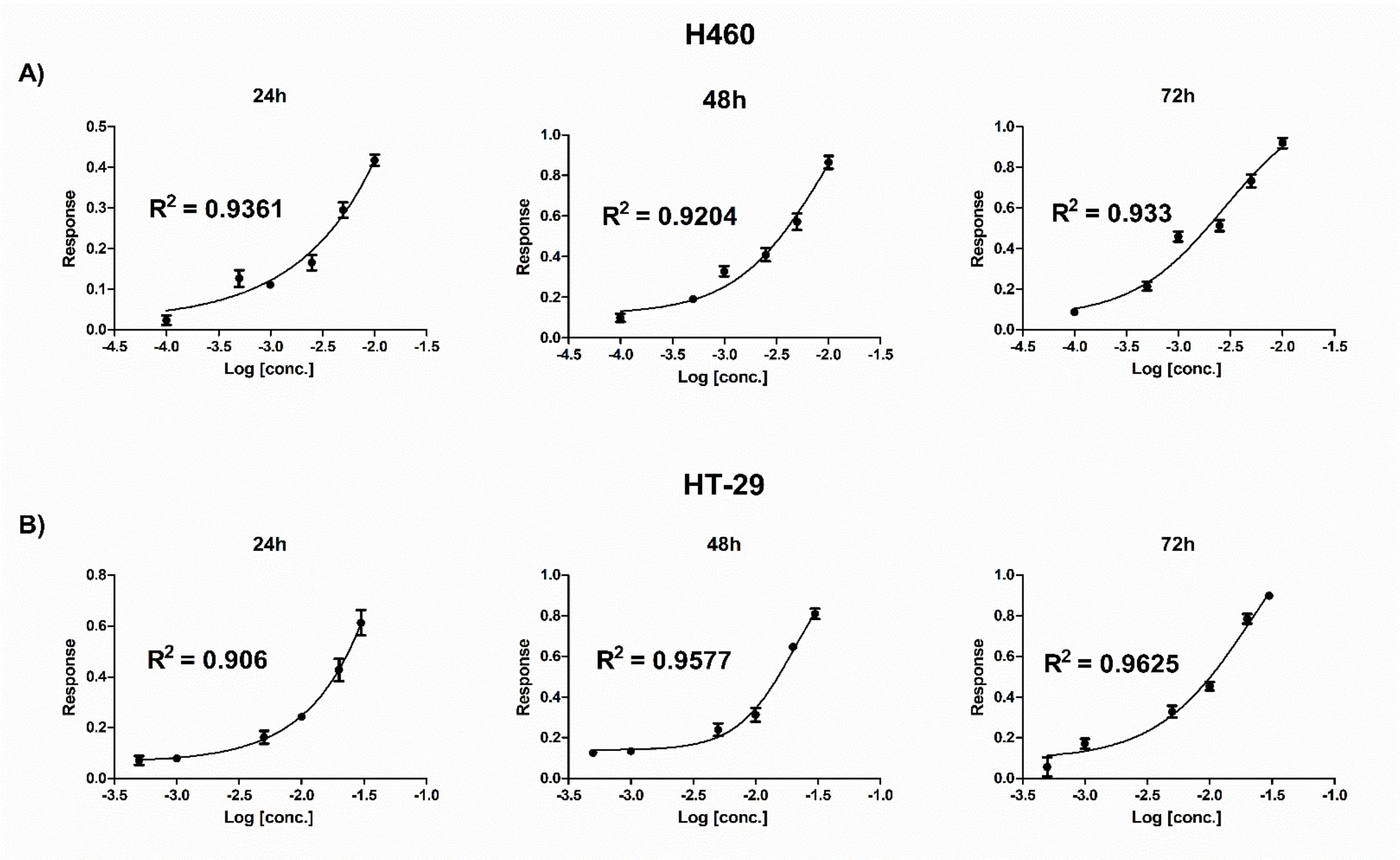

Figure S3. Non-linear regression dose-response curves for CIS in H460 **(A)** and HT-29 **(B)** at 24, 48, and 72 h. Response ratio is plotted as a function of log- concentration. Curves represent non-linear fits generated using a variable-slope sigmoidal dose-response model. Data points are presented as the mean ± SD from three independent experiments (n = 3). Goodness-of-fit (R^2) values for each curve are indicated within the respective panels.

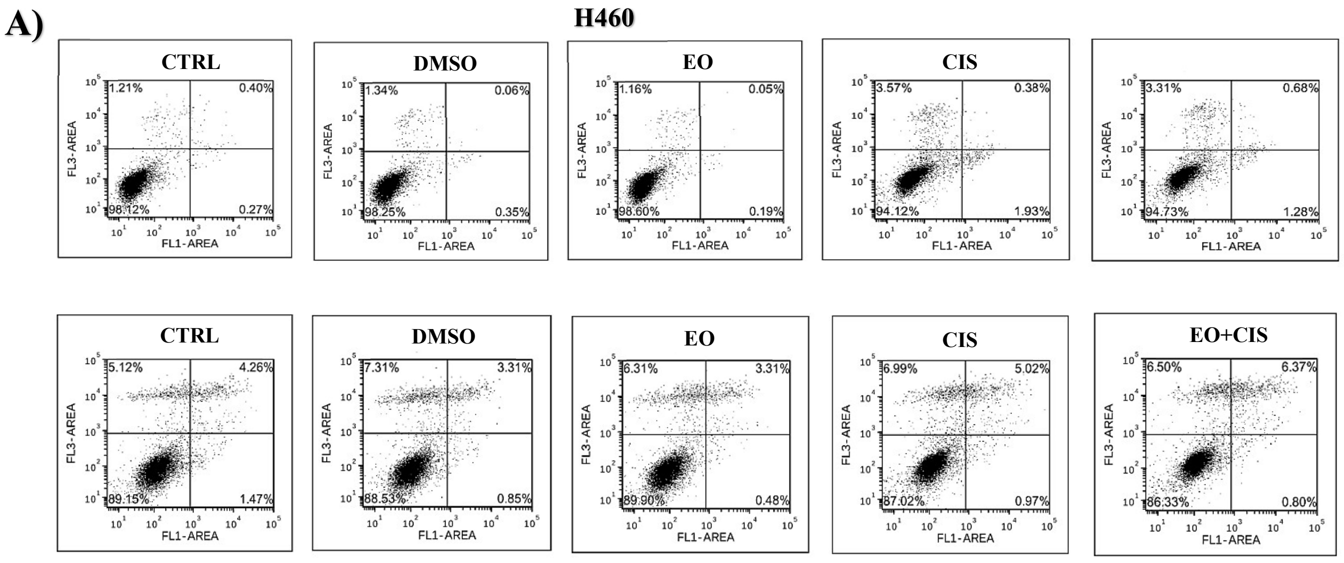

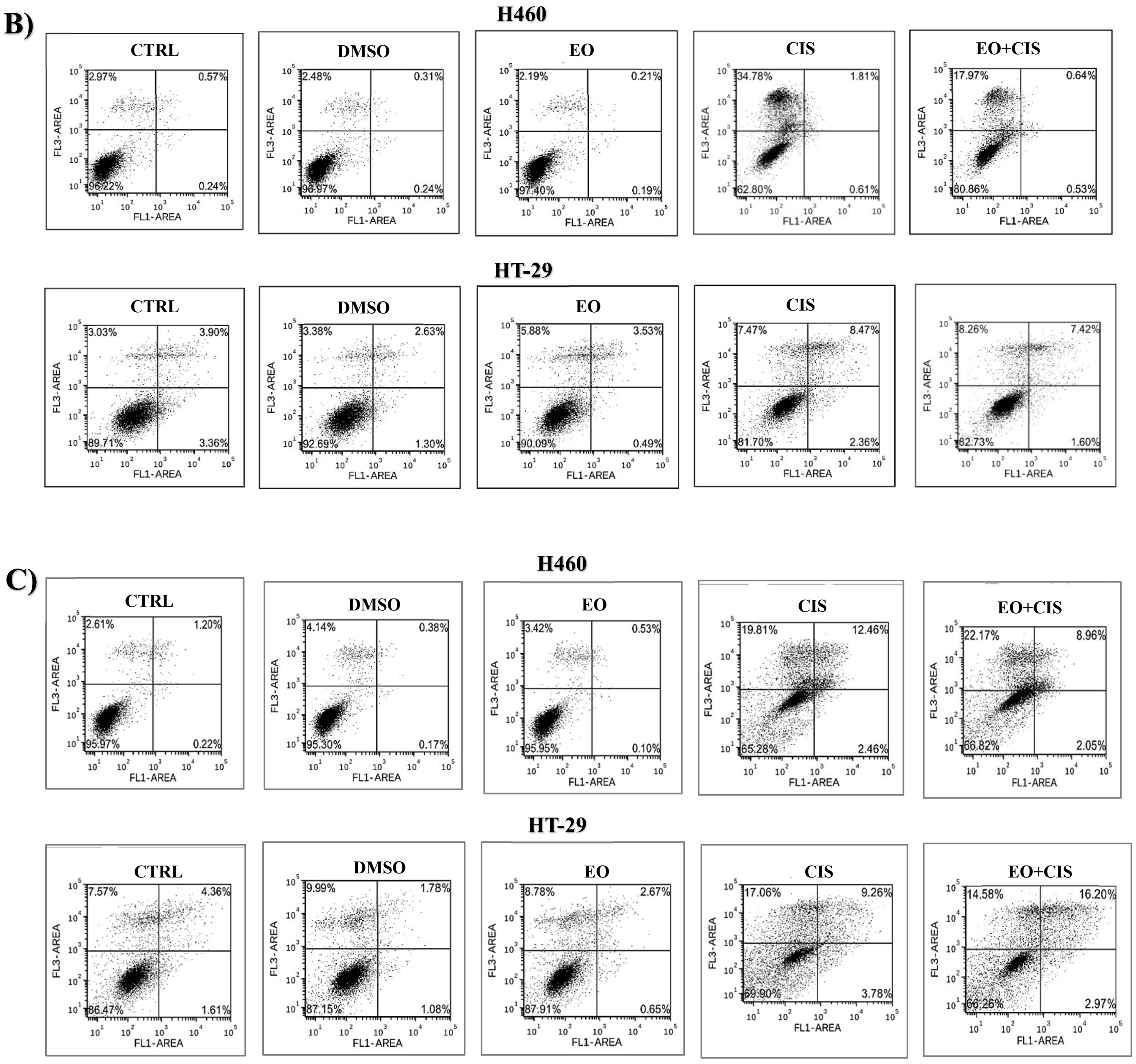

Figure S4. Representative flow cytometry dot plots of Annexin V-APC/PI dual-staining in H460 and HT-29 cell lines following treatment with EO, CIS, and their combination for 24 hours **(A)**, 48 hours **(B)** and 72 hours **(C)**. Quadrants represent viable cells (lower left, Annexin V^-/PI^-), early apoptotic cells (lower right, Annexin V^+/PI^-), late apoptotic cells (upper right, Annexin V^+/PI^+), and necrotic cells (upper left, Annexin V^-/PI^+). The Data reflect representative profiles from three independent experiments ($n = 3$).

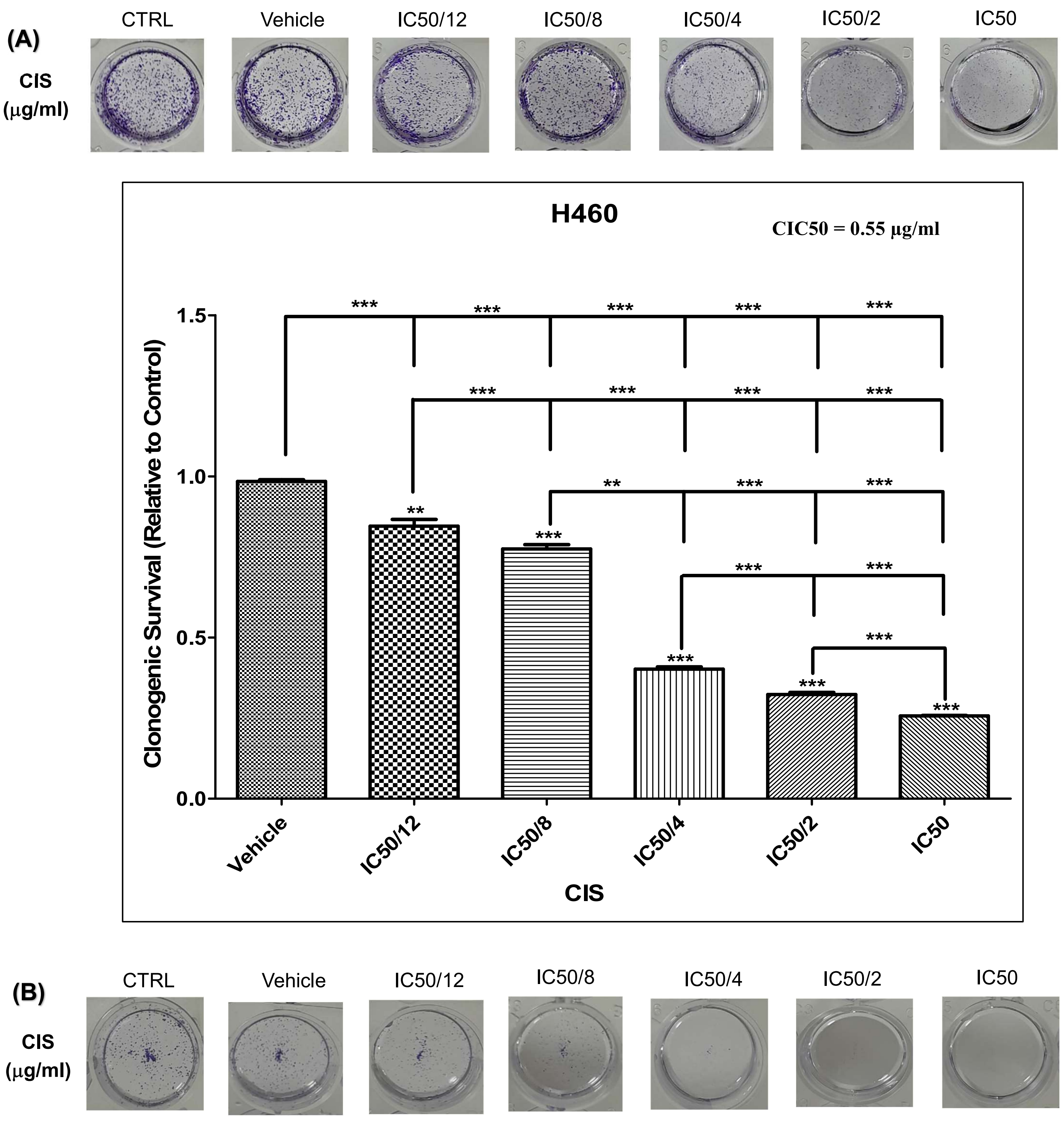
(A)
CIS (μg/ml)
CTRL
Vehicle
IC50/12
IC50/8
IC50/4
IC50/2
IC50
H460
CIC50 = 0.55 μg/ml
Clonogenic Survival (Relative to Control)
1.5
1.0
0.5
0.0
***
**
Vehicle
IC50/12
IC50/8
IC50/4
IC50/2
IC50
CIS
(B)
CIS (μg/ml)
CTRL
Vehicle
IC50/12
IC50/8
IC50/4
IC50/2
IC50

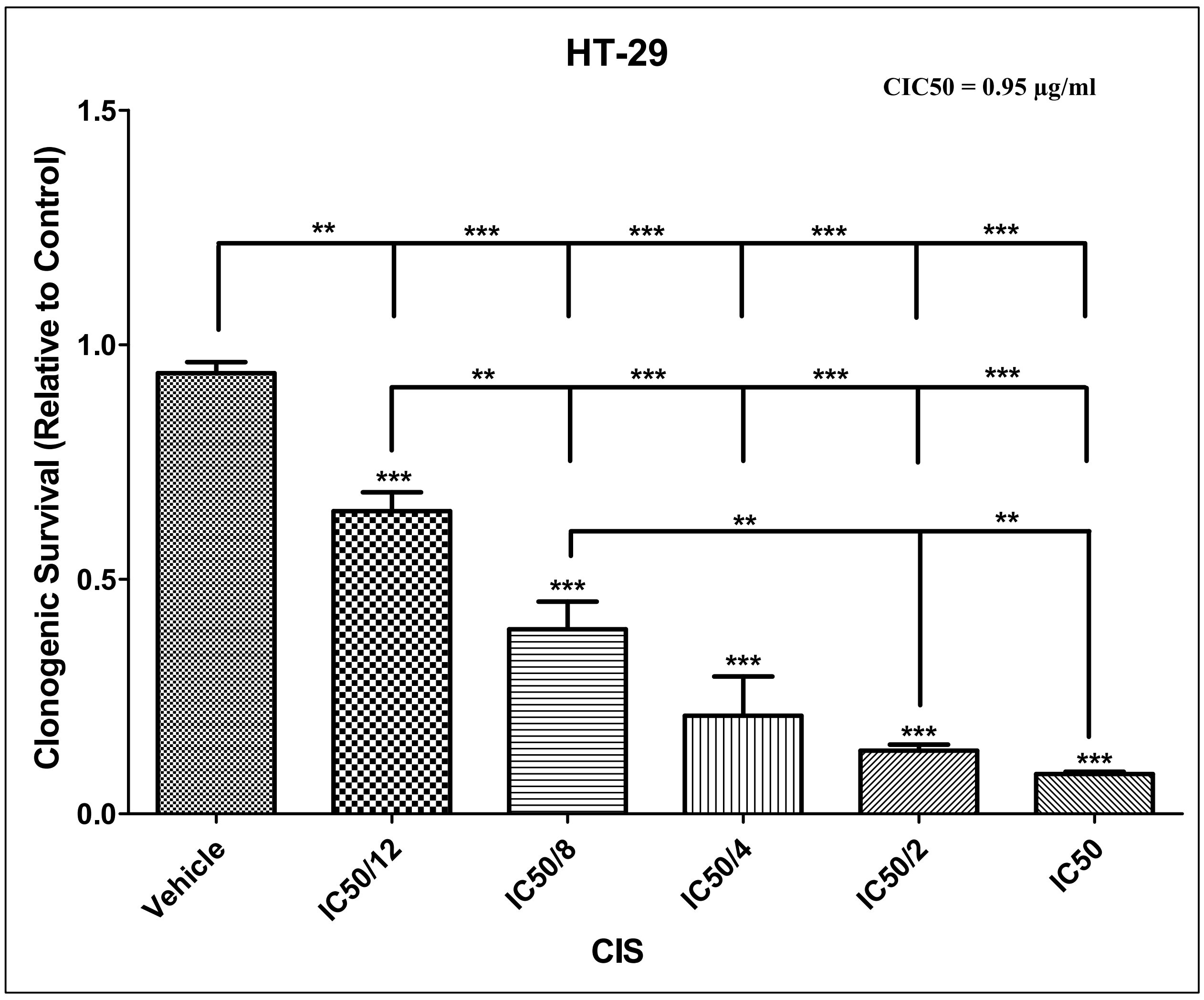

Figure S5: Dose-dependent effect of CIS on colony biomass of H460 (A) and HT-29 (B). Data analysis for CIC50 determination (Dose inhibiting 50% of colony biomass formation) was conducted by fitting % Response=f(log[conc.]) via GraphPad Prism 5.00. Data presented is the mean ± SD from three different experiments. Statistical significance was conducted via Tukey post-hoc analysis following One-way ANOVA. Asterisks on bars represent significance relative to the control. Asterisks on connecting lines represent significant differences across conditions (including the vehicle group). (*), (**), and (***) correspond to *p*< 0.05, 0.01, and 0.001, respectively.
